# Highlights from the 2012 International Symposium on HIV & Emerging Infectious Diseases (ISHEID): from cART management to the search of an HIV cure

**DOI:** 10.1186/1742-6405-9-23

**Published:** 2012-08-01

**Authors:** Alain Lafeuillade, Vicente Soriano, Marie Suzan-Monti, Mario Stevenson, Jacques Izopet, Hans-Jürgen Stellbrink

**Affiliations:** 1 Department of Infectious Diseases, General Hospital, Toulon, France; 2 Department of Infectious Diseases Hospital Carlos III, Madrid, Spain; 3 INSERM, U912 (SESSTIM), Marseille, France; 4 Aix Marseille Université, IRD, UMR_S912, Marseille, France; 5 ORS PACA, Observatoire Régional de la Santé Provence Alpes Côte d'Azur, Marseille, France; 6 Miller MedicalSchool, University of Miami, Miami, FL, USA; 7 Laboratoire de Virologie, Institut Fédératif de Biologie, Hôpital Purpan, Toulouse, France; 8 Infektions Medizinisches Centrum Hamburg Study Center, Hamburg, Germany

**Keywords:** HIV pandemic, HIV cure, HIV reservoirs, Antiretroviral therapy, HCV coinfection, Access to care, New anti-HCV drugs

## Abstract

The 2012 International Symposium on HIV and Emerging Infectious Diseases (ISHEID) provided a forum for investigators to hear the latest research developments in the clinical management of HIV and HCV infections as well as HIV-1 reservoirs and cure research. Combined anti-retroviral therapy (c-ART) has had a profound impact on the disease prognosis of individuals living with HIV-1 infection. However, although these anti-retroviral regimens are able to reduce plasma viremia to below the limits of detection for sustained periods of time, there is a rapid recrudescence in plasma viremia if treatment is interrupted. Therefore, despite this potent anti-retroviral suppression, HIV-1 is able to persist within the infected individual. The main 2012 ISHEID theme was, hence “searching for an HIV cure”. In this report we not only give details on this main topic of the 2012 ISHEID but also summarize what has been discussed in the areas of HIV epidemiology, access to care, antiretroviral therapy management and recent progress in the therapy of HCV infection in patients with HIV.

## Introduction

The 2012 International Symposium on HIV and Emerging Infectious Diseases (ISHEID) was held in Marseilles, France, on May 23–25. This congress attracted more than 1,000 participants from all over the world (70% outside France) and allowed detailed discussions around current issues in HIV research and care. In this summary, we focus on the epidemiological aspects, the study of HIV reservoirs and the search for an HIV cure, combined antiretroviral therapy (c-ART) management in 2012 and new therapies for patients coinfected by HCV.

### Update on HIV epidemic, prevention and progress towards universal access

Anna Mia Ekström reported recent changes in HIV incidence, with a tendency to stability or decrease, except for Eastern Europe and Central Asia [1]. A HIV concentrated epidemic developed in Bulgaria, among most at-risk groups (IVDUs, MSM, prisoners and people from Romania) [2]. Annual new infections dropped by 21% since 1997, but in 2010 new infections (2.7 millions) still exceeded AIDS-related deaths (1.8 millions), 97% of them occurring in low- and middle-income countries (LMIC) [3]. Three points were discussed: i) the difficulty to measure HIV incidence using current methodologies since HIV prevalence became a poor estimate of incidence due to different factors including access to c-ART and variable retention in c-ART programs affecting survival; ii) c-ART coverage is not a sufficient guide to measure the success of scaling up to treatment since the percentage of lost-to-follow up patients (LTFU) 48 months after c-ART initiation varies from 20 to 45% in LMIC. The drop-out problem affected also the success of PMTCT programs, pointing the need to measure completion rather than enrolment into programs; iii) the real-effectiveness of c-ART programs in Sub-Saharan Africa is heavily impacted by health system factors resulting in a drop from 90% efficacy to 19% effectiveness.

Yves Souteyrand presented the WHO targets for 2015 and the challenges that need to be addressed [4], including increasing resources for HIV programs while reducing inequities in LMIC. The global resources allocated to HIV programs dropped from 16 to 15 billion USD for the first time in 2010. To achieve universal access to c-ART governments must be consistent with their engagements. Although global c-ART coverage was 47% by the end of 2010, geographic, demographic, socio-economic and vulnerable groups-related inequities produced large variations in coverage, from 10% in North Africa and the Middle East to 63% in Latin America and the Caribbean. Other key challenges consist in improving earlier access to treatment and sustaining long-term treatment. Beside the problem of LTFU, attention must be paid to retention in pre c-ART care where substantial LTFU is reported from HIV-testing to ART initiation [5]. HIV and STDs testing, CD4 cell counting and viral load at the 1^st^ visit in the HIV program in Mexico City resulted in 51% detection increase but 60% of newly HIV-diagnosed people were not linked to care [6].

The Treatment 2.0 framework [7] represents a comprehensive approach to achieve these goals, focused on 5 areas of actions: 1) optimize drug regimens; 2) improve access to a package of simple affordable point-of-care and other simplified diagnostics; 3) reduce treatment costs; 4) improve delivery systems; 5) mobilize communities in HIV prevention, treatment, and human rights-related issues. In the Karnataka province (India), coordination between government and civil society partners resulted in higher decentralization of ART delivery services and increase in HIV diagnoses [8], with positive impacts on reduction in LTFU, on adherence and on strengthening primary and secondary health care service, beneficial for program sustainability [9]. In 2010 in Armenia, 73% of HIV diagnosed people were linked to care, 46% started ART and 14.9% were LTFU. However, 57.7% of newly HIV-infected people were diagnosed lately, pointing the need to reinforce this program for efficient entry into care [10].

Another issue to achieve universal access concerns health policy decisions at governmental levels in the context of financial constraints. YazdanYazdanpanah asked how to best utilize available resources in LIC or HIC. Cost-effectiveness analyses (CEA) are tools to assist decision makers in choosing from among competing alternatives, in situations of uncertainty and limited resources for prioritizing the use of health care services, but they are one component among others including fairness, ethics, and political issues. Most CEAs focus on more effective intervention that are often more expensive and allow to consider whether the additional benefit is worth the additional cost. They can be used to consider optimal timing of ART initiation. Studies in South-Africa [11] and France [12] showed that earlier treatments may significantly reduce HIV burden and costs. CEAs were used to evaluate HIV testing strategies [13] or pre-exposure prophylaxis (PrEP) interventions, showing that high PrEP efficacy in high HIV incidence populations is cost-saving and cost-effective [14].

HIV treatment as prevention (TasP), contrarily to PrEP strategies, is no more controversial today. Based on results from numerous studies demonstrating TasP as a potent tool to reduce HIV transmission, Joep Lange stated that implementation of TasP should not await further efficacy trials and need national plans for TasP roll-out. Although designated as the breakthrough of year 2011[15], TasP will not be enough to end the HIV epidemic [16]. The spectrum of engagement in HIV care in USA showed that only 19% of HIV-infected people had an undetectable viral load [17]. The challenge is to keep everybody in treatment to lower HIV transmission rate and decrease incidence, but also to treat earlier to maximize individual and public health benefits. Moreover TasP should be scaled up in conjunction with other effective HIV prevention interventions.

Mark Wainberg discussed results and controversies about PrEP strategies that appeared to be the hottest point of debate of this meeting [18]. Will the use of ART before sexual exposure prevent HIV acquisition? Efficacy was largely demonstrated in many *in vitro* experiments and in monkey models. Human efficacy trials, based on Tenofovir® treatment versus placebo design, produced inconsistent results.Of the 5 trials using oral daily PrEP, 2 failed to show a benefit (VOICE, Fem-PrEP) while iPrex, TDF-2 and Partners Prep trials reported 44 to 75% reduction of HIV transmission, respectively. The Caprisa trial, based on vaginal Tenofovir® gel, resulted in 39% transmission reduction [19]. Adherence was key issue since detectable drug levels strongly correlated with prophylactic effect. Several questions were raised: is daily PrEP high adherence achievable? Although few resistant viruses were described in these trials, the question of emergence and spread of resistances must be addressed. How to exclude acute infection before starting any PrEP? Is there a risk of change in behavior that could off-set the benefit of PrEP? Similar safer sex practices, fewer sexual partners and no difference in STDs acquisition were observed among iPrex participants. Is oral PrEP safe enough? Adverse events were significantly more frequent in the TDF/FTC arm in the Fem-Prep trial. Evidence for safety needs to be strongly addressed. The PrEP strategies raised rightful fears considering the current situation of the HIV epidemic: treatments are for those who need them; other prevention strategies are much more efficient; they will increase health care-related costs and re-allocate resources from research or prevention fields.

Intermittent PrEP strategies could represent an alternative to daily PrEP. They are based on data from animal models and from the Caprisa trial where intermittent use of TDF gel was effective, whereas its daily use in VOICE was ineffective. They could induce a better adherence with a potentially better efficacy/safety ratio and could be more cost-effective than daily PrEP. This hypothesis will be tested in the ANRS IPERGAY trial [20]. There are not yet clear responses to these challenges and further research to evaluate PrEP strategies is necessary before concluding the debate.

### HIV reservoirs & the search of an HIV cure

Animal models are crucial for a better understanding of HIV persistence during therapy and for the development of novel therapeutic strategies in the quest of a cure for HIV/AIDS. While sterilizing cure is defined by complete eradication of HIV infected cells from the body, functional cure is defined by undetectable viremia without antiretroviral therapy, no CD4 loss, no immunological defects and no disease progression. Guido Silvestri reviewed the similarities between HIV and pathogenic SIV infection of macaques including chronic immune activation, mucosal immune dysfunction, microbial translocation and high levels of infection of central-memory CD4+ T cells [21]. These non-human primate models provide real opportunities for several reasons: (i) identity, dose, and route of virus challenge are known (ii) various clinical parameters such time of infection or duration of c-ART can be controlled (iii) active and persistent reservoirs can be fully characterized (iv) testing of “risky” interventions is possible. Longitudinal collections of blood/tissue, as well as elective necropsy are available for determining virologic and immunologic parameters. Among limitations, challenge viruses (SIV, SHIV) are not HIV and obtaining levels of virus suppression similar to these obtained in HIV-infected humans is sometimes hard to achieve. To conclude, Guido Silvestri discussed pros and cons of developing a standardized non-human primate resource and underlined the need of close collaboration between “monkey people” and “human people”.

The sources of HIV persistence in c-ART-treated individuals could arise from residual ongoing viral replication or resting CD4^+^ T cells (and/or other cellular reservoirs) harboring stably integrated, transcriptionally silent but replication-competent proviruses. Carine Van Lint reviewed the multiple mechanisms that control HIV latency at a molecular level [22]. Beside post-transcriptional blockade via inefficient viral mRNA transport and inhibitory miRNAs, several blockades occur at a transcriptional level. Strong preference for proviral integration in protein-encoding transcriptionally active genes has been demonstrated. Recent data indicate a modest preference for integration in the same transcriptional orientation as the host gene, suggesting that transcriptional interference could play a major role in the establishment and maintenance of HIV-1 latency through different mechanisms (enhancer trapping, promoter occlusion, steric hindrance). The chromatin organization of the provirus and the epigenetic control of the promoter region (histone acetylation and methylation, DNA methylation) are also important. However, the high degree of methylation of HIV-1 promoters from infected individuals receiving suppressive antiretroviral therapy was not confirmed in a recent study. The absence of inducible cellular transcription factors, such as NF-KB and NFAT, that are excluded from the nuclei of resting cells, the sequestration of P-TEFb in a HEXIM/75 K sn RNA-bound inactive form and the absence of Tat and Tat-associated factors are the other mechanisms that maintain HIV-1 latency.

**Figure 1:**
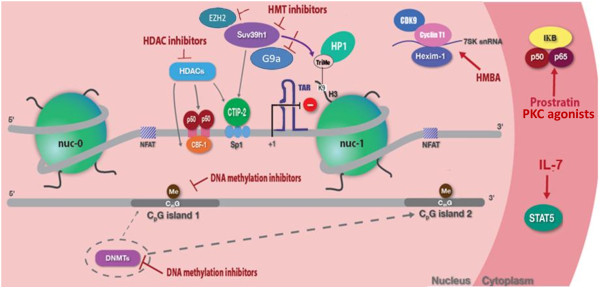
Different targets and drugs to reactivate proviral HIV-1 DNA from latency.

Several approaches can be used to reactivate viral latency with the expectation that new cell infections can be blocked *in vivo* by immune surveillance or antiretroviral drugs. IL7 or PKC agonists like prostratin can induce cellular transcription factors (STAT 5, NF-KB). HDACi (Histone deacyltransferase inhibitors) like SAHA (Vorinostat) promote histone acetylation, HMTi (Histone methyltransferase inhibitors) like chaetocin decrease histone methylation and DNA methylation inhibitors like decitabine decrease DNA methylation. HMBA induce the recruitment of the kinase complex P-TEFb (Cyclin T1/CDK9) to the HIV-1 promoter. Synergistic activation of HIV-1 expression by prostratin and vorinostat has been observed *in vitro*. Similarly, synergistic activation of HIV-1 expression by prostratin and HMBA has been obtained. Therefore one of the most promising strategies to target latent reservoirs could reside in combinations of several families of compounds (Figure1).

Tae-Wook Chun reviewed the main basic and clinical aspects of HIV reservoirs that were discovered during the past two decades [23]. Understanding the mechanisms by which HIV persists in the face of potent antiretroviral therapy is crucial for developing strategies to eliminate reservoirs and ultimately eradicate HIV. Homeostatic expansion of latent viral reservoir is an important issue but recent infections due to residual HIV replication, particularly in the gut lymphoid tissue, have to be taken into account. In addition, cell-to-cell spread of HIV permits ongoing replication despite antiretroviral therapy. The use of HDACi (such as vorinostat) for purging HIV in latently infected, resting CD4^+^ T cells from infected individuals on ART had a minimal impact compared to prostratin and anti-CD3. Recent data indicate that stimulation of patient CD8+ T cells with HIV gag peptides enhanced the cytotoxic T cell (CTL) responses and led to killing of vorinostat-treated cells. Therefore reactivation of viral latency *in vivo* is probably insufficient to accelerate the death of the reactivated cell and will require therapeutic vaccine to boost CTL responses in patients undergoing purging protocols to eliminate the latent reservoir. Gene therapy based on zinc-finger nuclease-mediated elimination of HIV coreceptors and stem cell transplantation from CCR5 delta 32 homozygous donors can generate HIV-resistant CD4^+^ T cells but important challenges need to be addressed.

Romas Geleziunas [24] discussed progress in the search for small molecule activators of latent HIV. He described a primary cell, HIV latency assay modified from an original assay developed by Planelles of the University of Utah. In this assay, memory CD4^+^ T cells are generated from naive T cells by antibody and cytokine treatment and then infected with a luciferase encoding HIV-1 variant that undergoes a single cycle of infection. The assay exploits a codon optimized luciferase that increases the sensitivity of the reactivation assay. The assay has been miniaturized to 384 well plates and automated for high throughput screening employing ten thousand cells per 20 uL well and 20 nL of compound. The screening efforts have focused on HDACi. Histone acetylation of the HIV-1 LTR is believed to be a major mechanism through which transcription of the LTR is blocked and inhibitors of histone deacetylases have been demonstrated to activate latent HIV-1 *in vitro*. Vorinostat (SAHA) is an HDAC inhibitor and is a licensed drug for the treatment of cutaneous T cell lymphoma. Toxicity profiles for SAHA are well described and two clinical trials exploring the ability of SAHA to reactivate latent HIV *in vivo* are currently being carried out by David Margolis at the University of North Carolina and Sharon Lewin at the University of Melbourne. Preliminary studies from Margolis laboratory indicate that SAHA is able to reactivate latent HIV *in vivo*. Geleziunas described results with the HDACi, Romidepsin that is FDA approved for the treatment of cutaneous T cell lymphoma. Remarkably, Romidepsin is a thousand fold more potent then SAHA at inducing latent HIV and one thousand to twenty thousand more potent in inhibiting HDAC enzymatic activity. Romidepsin was able to activate HIV RNA production in twelve of thirteen HIV-1 infected subjects on suppressive c-ART. The average increase in viral RNA was approximately 8.6 fold. Therefore Romidepsin may be a better inducer of latent HIV *in vivo* and its favorable toxicity profiles would allow multiple dose studies. While this is an exciting development, an overarching question is whether the increases in viral RNA induced either by SAHA or Romidepsin will be sufficient to raise the amount of viral protein expression in reactivated cell to levels that lead to viral cytopathicity and/or elimination by cytotoxic T cells. Recent work from the Siliciano laboratory [25] indicates that after reversal of latency *in vitro*, reactivated resting T cells survived viral cytopathic effects even in the presence of autologous cytotoxic T lymphocytes from most patients on suppressive anti-retroviral therapy. Therefore, reactivation of viral latency may not be effective at eliminating the infected cell.

Susana Valente of the Scripts Research Institute in Jupiter, Florida described a novel compound that suppresses Tat-dependent HIV transcription [26]. Cortistatin A is a naturally occurring steroidal alkaloid isolated from marine sponges. Cortistatin A was originally shown to exert anti-proliferative activity against human umbilical vein endothelial cells and the laboratory of Valente became interested in Cortistatin A because it has also been described as a ligand of CDK11. Since the related CDK9 plays an essential role in activation of transcriptional elongation by HIV-1 Tat, Valente explored whether Cortistatin A may inhibit Tat activity.Cortistatin A was found to exert an inhibitory effect on acute HIV-1 infection at nanomolar concentrations and to repress transcription from the HIV promoter. Valente demonstrated that Cortistatin A binds to the basic domain of Tat and inhibits elongation from the HIV-1 promoter. Intriguingly, Cortistatin A was found to inhibit HIV reactivation from resting CD4 cells isolated from individuals on suppressive anti-retroviral therapy. Furthermore, Cortistatin A appeared to affect an irreversible effect on viral transcription since removal of Cortistatin A did not result in viral rebound. Therefore, Cortistatin A warrants serious consideration as an agent that can force HIV-1 into an irreversible state of latency. This approach would be fundamentally different from other approaches aimed at reactivating viral latency in order to purge infected cells.Cortistatin A may have the capacity to induce a permanent state of latency in cells of HIV-1 infected patients.

The laboratory of Tokameh Mahmoudi of Erasmus University Medical Center in Rotterdam provided evidence for the involvement of the Wnt signaling pathway in transcriptional regulation of the HIV-1 LTR [27]. A hallmark of Wnt signaling is stabilization of the transcriptional co-activator beta-catenin. Beta-catenin then regulates gene expression by binding members of the T-cell-specific transcription factor/lymphoid enhancer-binding factor 1 (TCF/LEF-1) family of transcription factors. Mahmoudi presented evidence that the HIV-1 LTR contains TCF/LEF binding sites and this prompted an investigation of the effect of Wnt activation on transcription of the latent HIV-1 LTR. LiCl, an inhibitor of Wnt signaling, was found to strongly synergize with valproic acid or SAHA in inducing reactivation of the latent HIV-1 LTR in a cell line model of HIV-1 latency. The level of activation of the latent LTR was found to correlate with the remodeling of NUC-1 by high resolution nucleosomal mapping. Regulators of the Wnt signaling pathway provide an additional strategy with which to promote reactivation of the latent LTR particularly in combination with HDACi.

Santiago Moreno summarized ongoing clinical trials aimed at reactivating viral latency *in vivo*[28].Disulfiram has been shown to activate HIV transcription in *in vitro* latency models of HIV. This prompted a pilot clinical trial involving 14 patients on c-ART with plasma viral loads below 50 copies/ml. Disulfiram was administrated for two weeks and the frequency of latently infected cells as well as residual viral viremia was measured during disulfiram administration. Overall, disulfiram was safe and well tolerated with few adverse events. Within two hours of initial administration of disulfiram, there was a significant increase in residual viremia from an average of 30 copies/ml to between 60 and 70 copies/ml. No significant changes in the size of the latent reservoir were observed at these intervals. Moreno then went on to summarize results from the SAHA trial being conducted by David Margolis at the University of North Carolina in Chapel Hill where SAHA administration resulted in a mean 4.8 fold increase in viral RNA expression in memory T cells. In contrast, there was no effect on residual viremia in this trial. Moreno also described a pilot clinical trial employing Bryostatin-1 in a Jurkat cell line model of HIV-1 latency. Bryostatin-1 has been show to reactivate HIV-1 through the PKC-dependent pathway and to down-regulate the expression of CD4 and CXCR4. Bryostatin-1 also synergizes with HDACi, VPA, and trichostatin A in reactivating HIV-1 latency. This has prompted a pilot clinical trial with a single dose of Bryostatin-1 followed by evaluation of latent cell reservoir size residual viremia and cell associated viral RNA. This pilot trial is ongoing.

In addition to the obstacles posed by reservoir of latently infected cells, a number of studies have raised the possibility that incomplete viral suppression may permit some degree of replenishment of the viral reservoir in patients on ART. In collaboration with Timothy Schacker and Ashley Haase at the University of Minnesota and Courtney Fletcher at the University of Nebraska, Mario Stevenson has been examining anti-retroviral therapy impacts on tissue reservoirs for HIV-1[29]. Gut associated lymphoid tissue (GALT) is the major site of viral replication in viremic patients since the majority of permissive lymphocytes are located within GALT. However, the impact of anti-retroviral therapy on this reservoir and viral activity in this reservoir in patients on suppressive therapy are not well understood.Stevenson and collaborators obtained lymphoid tissue for viremic individuals initiating c-ART. The virologic response was gauged by measuring unintegrated forms of viral DNA including linear forms and episomal forms (2-LTR circles). Within several weeks of initiating therapy plasma viremia fell to undetectable levels in all individuals. Despite this, unintegrated viral genomes were poorly resolved by one month and six months post initiation of anti-retroviral treatment and in some individuals levels of unintegrated DNA at 6 months exceed those at baseline or one-month intervals particularly in cells obtained from lymphoid tissue. Intracellular drug concentrations in cells from lymphoid tissue were found to be significantly lower than in cells from peripheral blood obtained at the same intervals. These preliminary data indicate discordance in the ability of anti-retrovirals to sequester within cells of lymphoid tissue and this raises the possibility that lymphoid tissue may provide conditions for *de novo* viral infection in the face of anti-retroviral suppression.

### Clinical and therapeutic aspects

As Roy Gulick [30] pointed out in his presentation on new antiretroviral drugs, the number of agents currently pursued in phase 2 and 3 clinical trials is limited, and among those only the attachment inhibitor BMS-663068 represents a novel class with a new mode of action. This leaves little hope that novel compounds will overcome the problems of current drugs in the near future.

Jean-Michel Pawlotsky [31] gave an overview over recently available anti-HCV agents and compounds currently under development, as well as major clinical trials and very promising pilot studies investigating these agents and interferon-free regimens. As David Back [32] pointed out in his presentation on drug – drug interactions, the two recently licensed HCV protease inhibitors have opened a new arena of pharmacokinetic interactions with antiretroviral compounds, which are partly very challenging. He also described several important pharmacokinetic interactions between e.g. parenteral or topical corticosteroids and clarified that cobicistat, the novel pharmacokinetic booster contained in preparations of elvitegravir, will pose new questions regarding compatibility with concomitant medication. He made clear that the optimal use of these drugs requires an understanding of their interactions by physicians treating co-infected patients, and that more studies are needed to improve the strategies of dealing with those interactions.

In his presentation on salvage therapy in 2012, Stefano Vella [33] made the point that very few subjects in the developed world currently reach a level of treatment failure that would require their classification as true salvage patients. However, in developing countries patients reach the point beyond which no promising options are available much earlier due to economic constraints. He argued that this gap should be closed by making options for resistant viruses available in these settings and defining an optimal sequence of regimens.

Jürgen Rockstroh [34] presented on the issues regarding timing of anti-viral therapy, choice of regimen, complications, and HCV-treatment in HCV co-infected patients. He clarified that the beneficial effects of early ART clearly outweigh potential liver toxicities, but that physicians should stay alerted to the possibility of yet unexpected untoward effects, such as the association of portal hypertension with didanosine use. The treatment armamentarium for HIV now offers chances for regimens with less interaction with novel anti-HCV compounds, so that HCV coinfection may be managed actively.

In his presentation on pharmacogenomics, Amalio Telenti [35] outlined the wide-spread use of pharmacogenomics in other fields of medicine, the advances in the HIV field, especially with respect to management of toxicity (e.g. efavirenz, atazanavir, abacavir) or cardiovascular risk. He criticized the exceptionalism with respect to the application of genetic analyses to clinical medicine, especially in Europe and made a strong case for moving forward with the implementation of these diagnostic tools.

The question of when to initiate c-ART was also addressed in a presentation by Cécile Goujard [36] about the ANRS C06 Primo cohort, in which 1450 patients with primary HIV infection (PHI) have been included until 12/2011, 52% of whom initiated antiretroviral therapy at PHI. Of note, 77% of those included at a CD4 count of <500/μL showed progression to <350 CD4 cells/μL after two years, whereas none of the patients included at >750 CD4 cells/μL showed progression. In this cohort, the level of HIV DNA was an independent predictor of progression. When comparing patients with transient c-ART initiated at PHI with the SEROCO cohort, there was no obvious impact of c-ART on time off therapy. She also described the interesting observation of 14 cases in whom HIV RNA was persistently undetectable for more than 12 months following interruption of ART initiated at PHI. They did not find an overrepresentation of the HLA class one alleles classically associated with elite control. The virological and immunological arguments for starting ART as early as possible were elucidated by Jean-Pierre Routy [37] and Joseph Wong [38]. They talked about viral persistence as the principal obstacle to cure and made the point that clinical trials investigating functional or sterilizing cure will have to include analytical treatment interruptions.

For some of the hot topics in clinical HIV medicine, the organizers selected the format of a pro and con debate. Magnus Gisslén and Hans-Jürgen Stellbrink discussed the relevance of HIV-associated neurocognitive disorders, Mark Wainberg and Mark Nelson took the pro and con positions for PrEP, and Alain Lafeuillade and Jean-Pierre Routy discussed the prospects of HIV cure. The audience witnessed very lively discussions. Their votes reflected some changes in opinion after the discussions and provided a valuable feedback to the presenters regarding the perception of their argumentation.

In a review of HCV treatment outcome in the Hepatitis session, Stanislas Pol [39] clarified the clinically very important point that remodeling of fibrosis following SVR is possible even in advanced liver disease, and that fibrosis reversal is associated with improvement of life expectancy. He clarified that ultrasound follow-up should be continued in patients with lack of fibrosis reversal following SVR, because progression to hepatocellular carcinoma may still occur in these subjects.

### Viral hepatitis

The burden of viral hepatitis is enormous worldwide. Hepatitis B virus (HBV) and HCV are by far the most frequent agents of chronic viral hepatitis. Chronic HBV infection affects 350 million people, being the highest prevalence found in Asia. Of note, superinfection with the hepatitis delta virus (HDV) in chronic HBsAg + carriers leads to chronic hepatitis delta, which is the most aggressive form of viral hepatitis. A study from Taiwan that examined 1,186 HBsAg + patients found anti-HDV antibodies in up to 55% of 341 HIV-HBV coinfected individuals, stressing on the poor outcome of these patients. Delta superinfection was associated with intravenous drug use as well as with HCV coinfection [40]. Interestingly, HDV genotype IV predominated among injecting drug users whereas genotype II was the most frequent variant in other individuals with hepatitis delta in Taiwan.

Around 2% of the world population is infected by HCV, which represents 175 million people. The proportion of persons aware of their HCV infection is estimated to be below 40% in the United States and only rise up to 60% in France. Therefore, active HCV diagnosis remains crucial at a time that treatment for chronic hepatitis C has become much more successful following the arrival of directly acting antivirals (DAA). As in HIV, proper strategies pursuing “*test and treat*” should now be implemented for hepatitis C.

There are several reasons to explain why hepatitis C therapy in HIV-infected individuals attracted so much attention at ISHEID 2012. First, overall one third of HIV+ individuals are coinfected with HCV, being this rate disproportionately higher among IVDUs or persons who acquired the virus from contaminated blood products (i.e., hemophiliacs). Second, the way the new hepatitis C antiviral agents are used reminds much what we are already familiar in HIV therapeutics, considering variables such as viral load, drug resistance, geno/subtypes, viral kinetics, combination therapy, etc. [41]. Third, the pharmaceutical companies that are developing DAA are almost the same that already are marketing antiretroviral agents [31]. So, broad overlap exists between HCV and HIV at multiple areas. Ultimately, a shift in prescribers of the new HCV medications should be expected in many places moving from classical gastroenterologists to infectious diseases doctors.

Despite the previous considerations, significant biological features distinguish HIV from HCV, which translate into clinical and therapeutic differences [31]. First, in contrast with HIV there is no integration of the HCV genetic material into the chromosomes of infected cells during the virus replication cycle. This explains why HCV can be eradicated from the infected carrier whereas there is no elimination feasible for HIV even in long-term suppressed persons on antiretroviral therapy [42]. Second, the genetic variability in HCV is much greater than in HIV, accounting for significant differences in HCV susceptibility to antiviral agents across distinct HCV genotypes and subtypes. For instance, HCV subtype 1a responds less well to many DAA than HCV subtype 1b. Moreover, selection of drug resistance mutations to HCV protease inhibitors occurs at different positions in HCV subtypes 1a versus 1b [43].

The management of chronic hepatitis C in the setting of HIV infection is particularly challenging, being drug-drug interactions, overlapping toxicities and difficulties associated to polymedication amongst the most troublesome [41]. Both first-generation HCV protease inhibitors boceprevir and telaprevir reduce significantly exposure to HIV protease inhibitors but atazanavir for which this effect is lower. Likewise, significant drug interactions occur between telaprevir/boceprevir and HIV non-nucleoside polymerase inhibitors nevirapine and efavirenz ( Additional file 1: Table S1). However, rilpivirine and etravirine do not interact significantly with DAA and therefore can be safely co-administered [32]. The same applies to raltegravir, which the most preferred third antiretroviral agent for HIV-HCV coinfected patients planned to be treated with DAA. Moreover, the good safety liver profile of this drug makes it particularly attractive for HIV-infected individuals with underlying liver disease [34].

**Figure 2:**
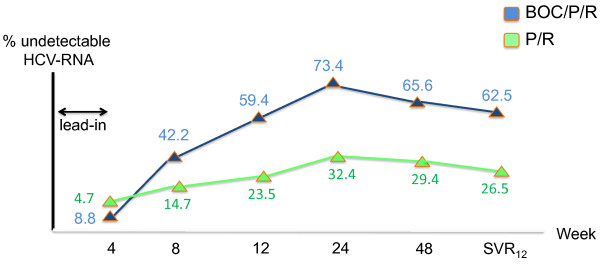
**Treatment of chronic hepatitis C withBoceprevir in HIV/HCV coinfected patients.** P: pegylated interferon, R: ribavirin, SVR_12_: sustained virological response at 12 weeks.

The results so far available with boceprevir and telaprevir in HIV/HCV-coinfected patients were deeply discussed at ISHEID [41]. In the phase 2 boceprevir study, a total of 98 HCV genotype 1 patients were randomized to receive either triple therapy or standard of care (peginterferon-ribavirin) for one year. Two thirds of patients were infected with HCV subtype 1a. Seven patients experienced viral breakthrough during therapy, 3/64 in the boceprevir arm and 4/34 in the control arm. Severe anemia developed in 5% of patients assigned to boceprevir and in 3% of controls. Erythropoietin was prescribed in 38% of patients on triple therapy and in 21% of controls. The rate of sustained virological response 12 weeks after completion of therapy (SVR_12_) was 61% versus 27%, respectively. Figure2 records the proportion of patients with undetectable HCV-RNA in the two study arms at different time points. Although all patients were on antiretroviral therapy (mostly HIV protease inhibitors), a similar proportion of patients on boceprevir (3/64) and controls (4/34) experienced HIV-RNA rebounds during hepatitis C therapy.

**Figure 3:**
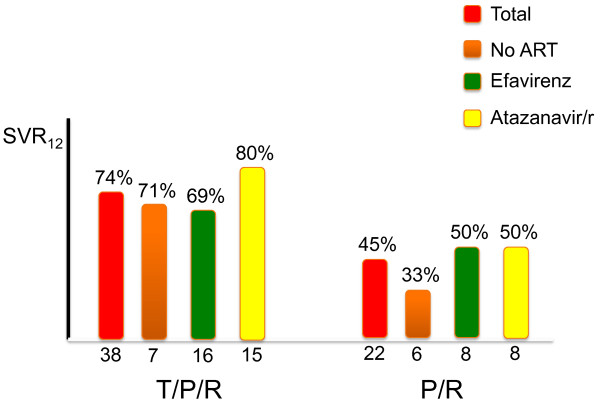
**Treatment of chronic hepatitis C with Telaprevir in HIV/HCV coinfected patients.** P: pegylated interferon, R: ribavirin.

In the phase 2 telaprevir trial (Study 110), 60 HIV-infected patients coinfected with HCV genotype 1 were randomized to receive either triple therapy or standard of care (peginterferon plus ribavirin). Of note, telaprevir only was given for the first 3 months of therapy, but all patients received peginterferon-ribavirin for one year. Thirteen patients did not receive antiretroviral therapy. Of the rest, 24 were on efavirenz and 23 on atazanavir/r. Early discontinuations due to side effects occurred in 3/38 patients on telaprevir and 0/22 in the control arm. Rash occurred in 13 (34%) patients on telaprevir and 5 (23%) controls. Anemia was recorded in 7 (18%) of patients on telaprevir and 4 (18%) of controls. SVR12 was 74% (28/38) on triple therapy versus 45% (10/22) on standard therapy. Relapses occurred in 1/32 (3%) of patients on telaprevir and 2/13 (15%) controls. Patients on atazanavir/r tended to respond better than those on efavirenz, although the numbers were too low to draw conclusions (Figure3). All patients on antiretroviral therapy kept on undetectable plasma HIV-RNA. The unexplained high rate of response in controls might suggest that the population enrolled in the 110 trial was somewhat unique and particularly prone to respond. In fact, the variation between triple therapy and standard of care was more pronounced for boceprevir than telaprevir (35% versus 29%, respectively).

Successful treatment of hepatitis C translates into definitive elimination of the virus from the body, and regression of liver fibrosis occurs even in persons who already had developed cirrhosis [44]. Cure has allowed unveiling that chronic HCV infection results in other abnormalities than hepatic inflammation and fibrosis. This is the case for metabolic abnormalities, including dislipidemia and insulin resistance, which ameliorate once chronic hepatitis C is successfully treated. Likewise, most autoimmune phenomena associated with persistent HCV replication tend to resolve once the virus is cleared with treatment. Finally, the recent appreciation of a negative impact of persistent immune activation by continuous HCV replication on body systems and organs has encouraged to treat chronic hepatitis C even in patients with minimal liver damage [45]. Again, learning from the consequences of uncontrolled viral replication in the HIV field is pushing the management of hepatitis C.

## Competing interests

The authors declare that they have no competing interests.

## Authors’ contributions

MSM wrote the epidemic, prevention and universal access section. AL, MS and JI wrote the HIV reservoirs & search for a cure section. HJS wrote the clinical and therapeutic section. VS wrote the hepatitis section. AL formatted the entire article. All authors read and approved the final manuscript.

## Supplementary Material

Additional file 1 **Table S1.** Pharmacological interactions between HCV protease inhibitors and antiretroviral drugs. Click here for file
