# Supplementary figures and images for: Highlights from the 2012 International Symposium on HIV & Emerging Infectious Diseases (ISHEID): from cART management to the search of an HIV cure
Source: AIDS Res Ther. 2012 Aug 1;9:23. doi: 10.1186/1742-6405-9-23 (PMC3472320; doi:10.1186/1742-6405-9-23)

## Slide 1
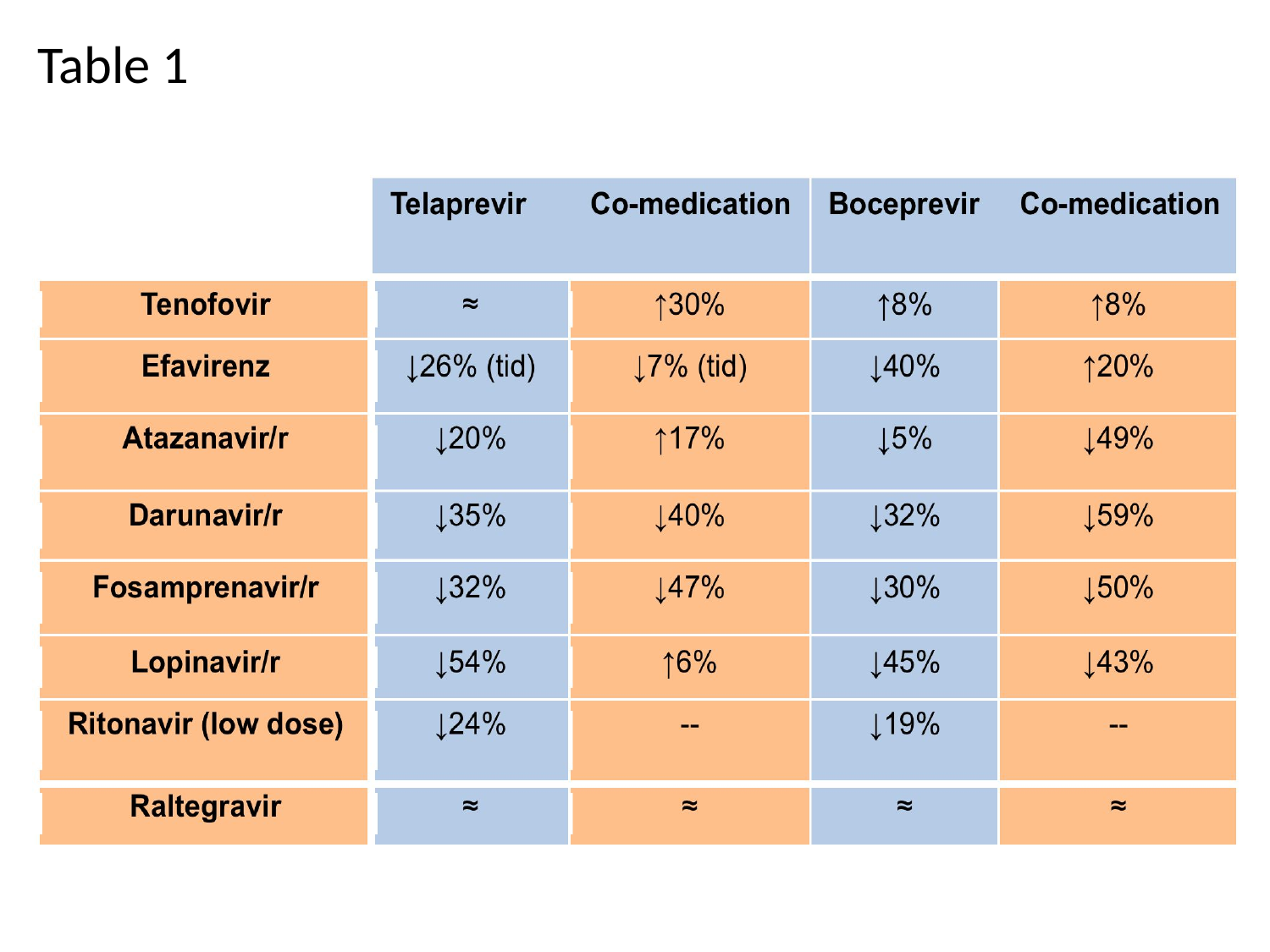

Table 1to reactivate HIV-1 from latency

Supplement: Additional file 1 — Table S1. Pharmacological interactions between HCV protease inhibitors and antiretroviral drugs. [file 1742-6405-9-23-S1.pptx]
